# Zebrafish Models for Dyskeratosis Congenita Reveal Critical Roles of p53 Activation Contributing to Hematopoietic Defects through RNA Processing

**DOI:** 10.1371/journal.pone.0030188

**Published:** 2012-01-27

**Authors:** Ying Zhang, Kenji Morimoto, Nadia Danilova, Bo Zhang, Shuo Lin

**Affiliations:** 1 Laboratory of Chemical Genomics, Shenzhen Graduate School, Peking University, Shenzhen, China; 2 Key Laboratory of Cell Proliferation and Differentiation of Ministry of Education, Center of Developmental Biology and Genetics, College of Life Sciences, Peking University, Beijing, China; 3 Department of Molecular, Cell and Developmental Biology, University of California Los Angeles, Los Angeles, California, United States of America; Tulane University Health Sciences Center, United States of America

## Abstract

Dyskeratosis congenita (DC) is a rare bone marrow failure syndrome in which hematopoietic defects are the main cause of mortality. The most studied gene responsible for DC pathogenesis is *DKC1* while mutations in several other genes encoding components of the H/ACA RNP telomerase complex, which is involved in ribosomal RNA(rRNA) processing and telomere maintenance, have also been implicated. GAR1/nola1 is one of the four core proteins of the H/ACA RNP complex. Through comparative analysis of morpholino oligonucleotide induced knockdown of *dkc1* and a retrovirus insertion induced mutation of *GAR1/nola1* in zebrafish, we demonstrate that hematopoietic defects are specifically recapitulated in these models and that these defects are significantly reduced in a *p53* null mutant background. We further show that changes in telomerase activity are undetectable at the early stages of DC pathogenesis but rRNA processing is clearly defective. Our data therefore support a model that deficiency in *dkc1* and *nola1* in the H/ACA RNP complex likely contributes to the hematopoietic phenotype through *p53* activation associated with rRNA processing defects rather than telomerase deficiency during the initial stage of DC pathogenesis.

## Introduction

Dyskeratosis congenita (DC) is a rare bone marrow failure syndrome associated with abnormal skin pigmentation, nail dystrophy, mucosal leukoplakia, pulmonary fibrosis, and an increased susceptibility to both hematopoietic and solid cancers [1]. 85% of DC patients experience bone marrow failure that accounts for 80% of all DC-related mortality [2]. Discovery that the telomerase complex gene *DKC1* was mutated in a subset of DC patients provided the first insight into a potential mechanism [3], [4].

*DKC1* encodes dyskerin, a pseudouridine synthase that complexes with box H/ACA small nuclear RNAs involved in posttranscriptional modification of ribosomal RNA (rRNA) through conversion of uridine (U) to pseudouridine (Y). Mutations in the catalytic domain of dyskerin lead to Hoyeraal-Hreidarsson syndrome resulting in a severe form of DC including immunodeficiency, growth retardation, and microcephaly. Dyskerin is also associated with the RNA component of telomerase that contains an H/ACA RNA motif.

Telomerase is a multimeric ribonucleoprotein complex responsible for maintaining telomere length in cells whose incomplete lagging strand synthesis and oxidative DNA damage result in progressive shortening of replicated DNA. Telomere shortening is associated with aging and genomic instability whose impact is widespread-healthy individuals with shorter telomeres possess a higher lifetime incidence of cancers [5] and shortened telomeres are associated with diverse pathologies including psychiatric disease [6], cardiovascular disease [7], idiopathic pulmonary fibrosis [8], and diabetes [9].

The telomerase complex consists of the transcriptase subunit TERT, the rRNA pseudouridylation dyskerin subunit adjoined to NOP10, NHP2, and GAR1, and the hTR rRNA (encoded by TERC) providing the template for reverse transcription. The telomere elongation and replication process is then continued by the shelterin complex.

At present, approximately 50% of DC patients have an identified mutation in one of eight genes involved in the telomerase complex [DKC1, TERC (encoding hTR), TERT, NHP2, NOP10], the Cajal body localizing co-factor TCAB1 [10], [11], the relatively unknown gene C16orf57 [12], or the shelterin complex (TINF2) [13].

*NOLA1* encodes GAR1p, a small nucleolar ribonucleic protein (snoRNP) that is critical for yeast 18 S rRNA maturation [14] and pseudouridylation of other precursor rRNAs [15]. It forms a complex with DKC1, NHP2, and NOP10; however, there are no reported *NOLA1* mutations in any human patients to date. Whereas knockdown of telomerase complex genes *DKC1* and *NOP10* result in a subsequent decrease in *TERC* expression, knockdown of GAR1p does not reduce *TERC* expression [16] suggesting that its critical role in rRNA maturation may involve non-telomerase complex associations.

Despite the clear association between DC patients and shortened telomere lengths, it remains unclear if shortened telomeres are the sole driver behind the disease phenotype. DC patients with *DKC1* and *TINF2* mutations typically present at younger ages and with more physical exam abnormalities than patients with *TERC* or *TERT* mutations yet there is no difference in telomere lengths between these subgroups [17]. Recently a subset of six DC patients harboring mutations in *C16orf57* all had normal telomere lengths despite severe disease penetrance at a young age [12]. It is therefore possible that other pathways aside from telomere maintenance are responsible for the disease phenotype.

Other bone marrow failure syndromes such as Diamond Blackfan Anemia(DBA) have been associated with *p53* pathway activation [18]. Up-regulation of *p53* has been reported in *Dkc1*-deficient mouse hepatocytes [19], suggesting that *p53* may be involved in the pathogenesis of DC as well.

We utilized a morpholino oligonucleotide (MO) knockdown approach to study the mechanism(s) by which the DC-associated gene *DKC1* results in hematopoietic stem cell failure. To further understand H/ACA RNPs complex interactions and its role in DC-related hematopoietic failure we also took advantage of a retroviral insertional mutation of *nola1*. We report that both models result in reduced hematopoiesis, increased *p53* expression, and defective ribosomal biogenesis all without detectable changes in telomerase function. These data suggest involvement of a telomerase-independent mechanism by which hematopoietic failure manifests in dyskeratosis congenita patients.

## Results

### *Dkc1* morphant and *nola1* mutant show similar morphological abnormalities

**Figure 1:**
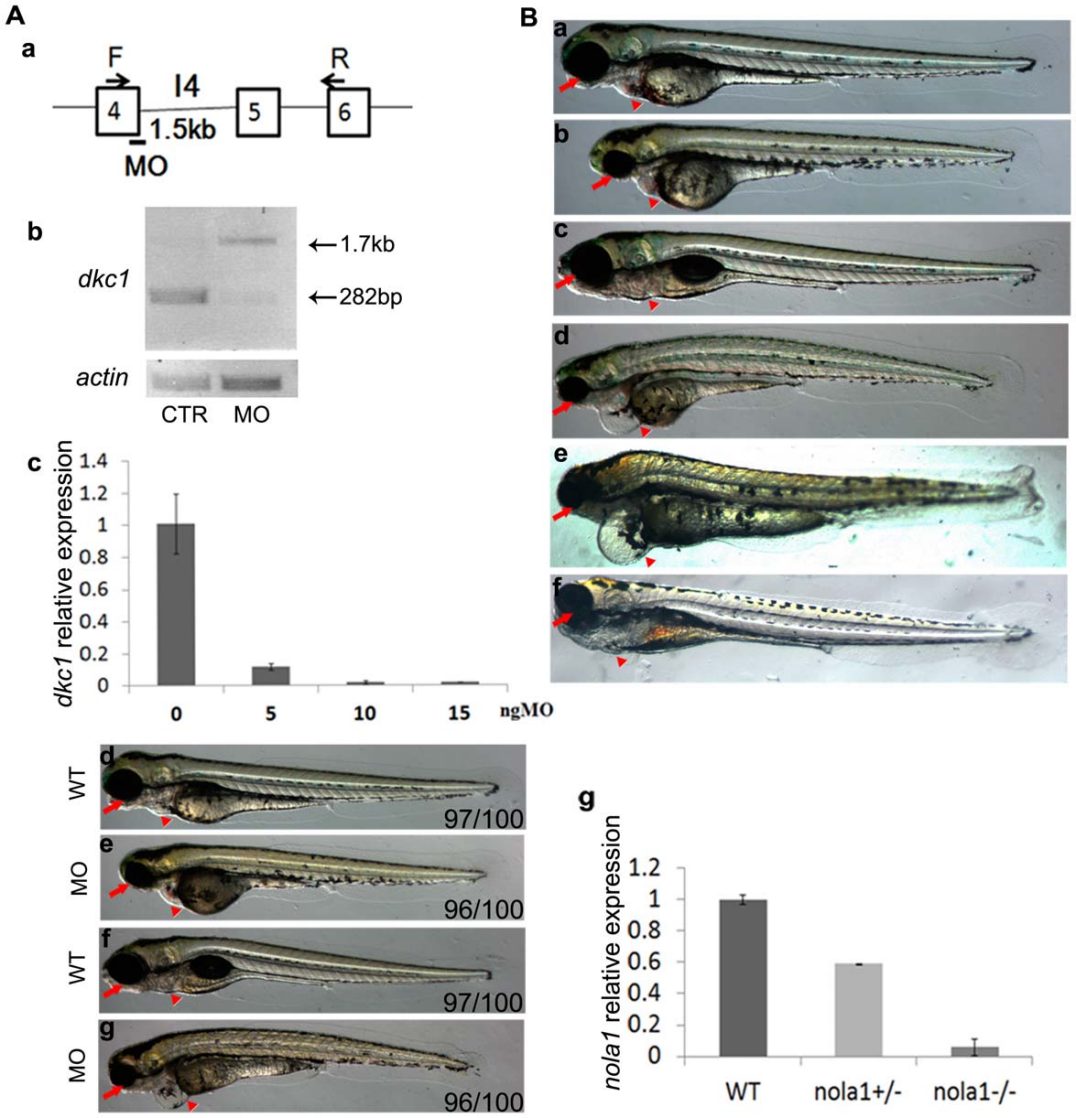
Molecular and phenotype analysis of *dkc1* morphant and *nola1* retroviral insertion mutatant of zebrafish. (**A**) Splicing MO targeting *dkc1* sequence caused the inclusion of intron 4 into mRNA (**a**). Semi-quantitive PCR data showed an increase of 1.5 kb in the PCR product in *dkc1* morphants (**b**). According to Real Time PCR result, expression of *dkc1* was diminished by more than 90% when high dose (15 ng) of MO was injected (**c**). Pictures of embryos at 3 dpf (**d** and **e**) and 5 dpf (**f** and **g**) showed smaller eyes and smaller head (red arrows in **d, e, f** and **g**) in *dkc1* morphants. Compared to wild type embryos, *dkc1* morphants developed edema, and had fewer red blood cells (red arrowhead in **d, e, f** and **g**). **d** and **f**: wild type controls; **e** and **g**: *dkc1* morphants. (**B**) A retroviral insertion in exon1 of the *nola1* gene led to the phenotype of smaller eyes, smaller head, and cardiac edema in *nola1* homozygous mutant. Red arrow indicated the smaller eyes and smaller head, and red arrowhead showed edema in *nola1* homozygous mutant at 3 dpf (**a** and **b**) and 5 dpf (**c** and **d**). Microinjection of *nola1* mRNA (**f**), but not *eGFP* mRNA (**e**) can rescue the mutant phenotype of *nola1* homozygous mutants at 5 dpf (red arrow and arrowhead in **e** and **f**). **a** and **c**: wild type siblings; **b**, **d**, **e** and **f**: *nola1* homozygous mutants. Expression of *nola1* was reduced more than 90% in *nola1* homozygous mutants (**g**). All the pictures of embryos are lateral view with anterior to the left.

To study the function of *dkc1* during embryonic zebrafish development we conducted a knockdown experiment with a splicing morpholino targeting the border between exon 4 and intron 4. This morpholino is predicted to cause inclusion of the 1.5 kb fourth intron into the mRNA transcript resulting in a truncated protein due to a pre-mature stop codon. Using primers spanning exons 4 and 6, morphants demonstrated a 1.5 kb larger *dkc1* transcript with correctly spliced *dkc1* transcript reduced to 10% of wild type levels (Fig. 1A b and c). These data demonstrate efficient knockdown of *dkc1*. From 3 days post-fertilization (dpf), *dkc1* morphants started to exhibit smaller heads and eyes, fail to develop the swim bladder, and develop edema (Fig. 1A e and g). Morphants died around 7 dpf.

We identified a zebrafish line (PKU #13771) carrying a pro-viral DNA integration in the first exon of *nola1* through a retrovirus insertion screen [20]. Protein sequence homology alignment revealed that *GAR1*/*nola1* is highly conserved among different species and human GAR1 and zebrafish nola1 protein sequences share 81.1% similarity and 79.0% identity. Genomic synteny relationship analysis also suggested that zebrafish *nola1* is the ortholog of the human *GAR1* (Fig.S2 A and B). Zebrafish *nola1* is ubiquitously expressed in most cells during early stages of development starting at 2 hours post-fertilization (hpf). Afterwards, *nola1* is mainly expressed in the brain and intestinal organs (Fig.S1). Homozygous *nola1* mutants shared similar morphologic changes with *dkc1* morphants (Fig. 1B b and d) and mutants died at 7 to 10 days post-fertilization (dpf). Genotyping confirmed that all embryos with the mutant phenotype were homozygous for the retroviral insertion (data not shown). Real time quantitative PCR (qPCR) demonstrated greater than 90% reduction in *nola1* mRNA expression in homozygous mutants (Fig. 1B g). The mutant phenotype was rescued by injection of *nola1* mRNA or *GAR1* mRNA (Fig. 1B f and Fig.S2C c), demonstrating the functional conservation of *GAR1/nola1*. Overall, functional studies of *dkc1* and *nola1*, two components of the H/ACA RNP complex, established that their functions were essential for zebrafish larval development and their deficiencies caused similar morphologic abnormalities.

### Both *dkc1* morphants and *nola1* mutants have hematopoietic defects

To determine if *dkc1* and *nola1* deficiencies in zebrafish would cause hematopoietic defects similar to DC patients, we analyzed multiple markers of primitive and definitive hematopoiesis by RNA whole-mount *in situ* hybridization. Prior to 3 dpf, no difference in circulation and hemoglobin staining was detected between *dkc1* morphants, *nola1* mutants and their wild type siblings (data not shown). Additionally, only minimal reduction in expression of the hematopoietic markers *scl* and *lmo2* was seen in *nola1* mutants at 24 hpf (data not shown). These data suggest that primitive hematopoiesis is, at most, minimally affected by *dkc1* or *nola1* deficiency.

**Figure 2:**
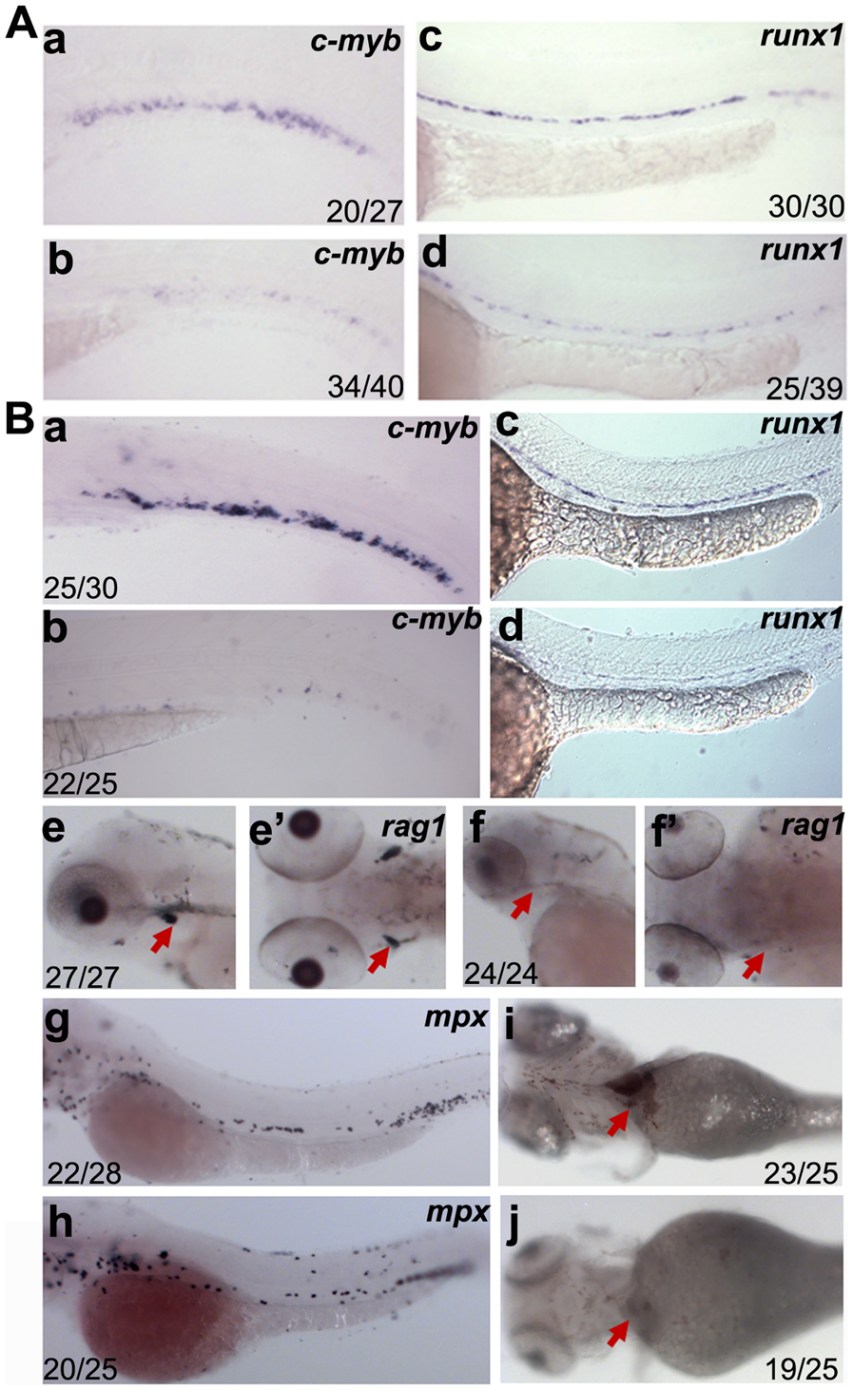
Analysis of hematopoietic defects in *dkc1* and *nola1* deficiency. (**A**) Expression of HSC marker genes was decreased in *dkc1* morphants. *C-myb* expression at 3 dpf (a and b); *runx1* expression at 30 hpf (c and d). (**B**) Number of HSC (a, b, c and d) and red blood cells (red arrow in i and j) at 4 dpf were significantly reduced in *nola1* mutants. *C-myb* expression at 3 dpf; *runx1* expression at 30 hpf. Granulocytes, marked by *mpx*, weren't affected or slightly less in *nola1* mutants at 3 dpf (g and h), while expression of *rag1*, a marker of lymphoid cells, almost disappeared in *nola1* mutants at 4 dpf (red arrow in e–f′). A a–d, B a–d, e, f, g and h are lateral view with anterior to the left, e′ and f′ are dorsal view with anterior to the left, and i and j are ventral view with anterior to the left.

However, expression of definitive hematopoietic stem cell (HSC) markers *runx1* and *c-myb* were significantly reduced in both *dkc1* and *nola1* deficient fish at 30 hpf and 3 dpf, respectively (Fig. 2A, Fig. 2B a, b, c and d). *Nola1* mutants also had greatly decreased hemoglobin as shown by o-dianisidine staining after 4 dpf (Fig. 2B i and j). This reduction persisted until 7 dpf (data not shown), indicating that the decrease in red blood cells was not caused by a delay of development. The decreased number of red blood cells may be due to the decreased production and/or decreased cell survival in definitive HSCs.

To explore whether *nola1* mutation resulted in reduced development in all definitive blood lineages, we examined granulocyte and lymphocyte development using *mpx* and *rag1* expression, respectively. Compared with wild type siblings, mutants' lymphoid cells were absent at day 4 (Fig. 2Be–2Bf′). In contrast, mutants had minimal change in the granulocyte marker *mpx* at 3 dpf (Fig. 2Bg–2Bh). These data indicate that definitive hematopoiesis is compromised to varying degrees by decreased *nola1* expression.

In conclusion, similar to what is observed in human DC patients, the deficiencies of *dkc1* and *nola1* in zebrafish result in defective hematopoiesis. We therefore view these zebrafish as potential models for DC.

### Hematopoietic reduction is mediated by *p53*

**Figure 3:**
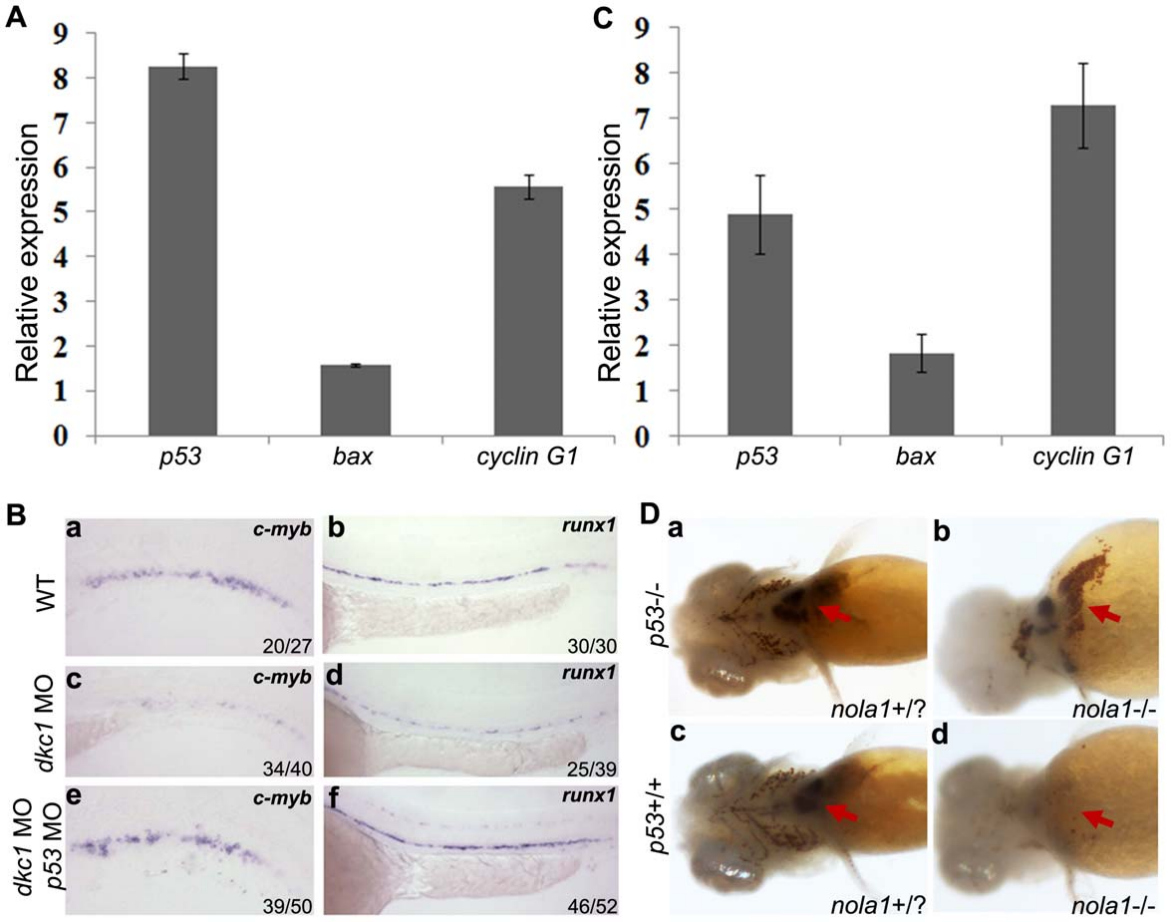
Activation of *p53* pathway and rescue of hematopoietic defects in zebrafish *dkc1* and *nola1* deficiency. (**A**) and (**C**) Expression of genes of *p53* pathway was up-regulated in both *dkc1* morphant (**A**) and *nola1* mutant (**C**). Expression level of wild type embryos of related genes was normalized as 1.0. (**B**) Knockdown of *p53* by injection of *p53* MO rescued the reduction of HSC in *dkc1* morphants (compare **c** with **e**, **d** with **f**). *C-myb* expression at 3 dpf (**a, c** and **e**); *runx1* expression at 30 hpf (**b, d** and **f**). (**D**) Red blood cells were restored in *nola1* and *p53* double mutatant embryos (red arrow in **a**–**d**). 25 embryos at 4 dpf were examined for each group. And the results shown here were representative for three independent experiments.

The *p53* pathway is activated in some animal models of congenital hematopoietic diseases, including zebrafish model of Diamond Blackfan Anemia (DBA) [18]. The *p53* network is also activated in telomerase deficient zebrafish [21]. However, the mechanism responsible for regulation of hematopoiesis by the *p53* pathway is not clear. Our qPCR results showed *p53* expression was increased greater than 4 fold in *dkc1* morphants and *nola1* mutants. *P53* downstream genes such as the pro-apoptotic gene *bax* and the cell cycle regulator gene *cyclin G1* also had significantly increased expression (Fig. 3A, C) suggesting that the *p53* pathway is activated with the H/ACA RNPs complex deficiency.

To determine if *p53* inhibition could rescue the hematopoietic defects seen in our models, we microinjected *p53* morpholino (blocking ATG translation) into *dkc1* morphants. Although the inhibition of *p53* did not rescue the morphological abnormalities seen in the *dkc1* morphants, down-regulation of *p53* did rescue *c-myb* and *runx1* expression in *dkc1* morphants (Fig. 3B). In a different experiment, we crossed *p53* null fish (tp53^M13K^) with *nola1* mutant fish and tested interaction of these two genes in a stable genetic mutant background. Detection of hemoglobin by the o-dianisidine staining on embryos of *p53* ^+/+^ *nola1* ^+/?^; *p53* ^+/+^ *nola1* ^−/−^; *p53* ^−/−^ *nola1* ^+/?^; *p53* ^−/−^ *nola1* ^−/−^ showed that the anemia observed in *nola1* mutants was rescued in the *p53* null background (Fig. 3D, arrows). Similarly, genetic mutation of p53 partially rescued *c-myb* expression (Fig.S3). Overall, our data suggest that the hematopoietic defects in *dkc1* morphants and *nola1* mutants are *p53*-dependent. The *p53* pathway may function in hematopoiesis by regulating the apoptosis and cell cycle of HSC and the differentiation of blood cell lineages.

### No telomerase activity defects are observed in *dkc1* and *nola1* deficient embryos but rRNA processing is inhibited

H/ACA RNP complex functions in both rRNA pseudouridylation and telomere maintenance [22]. To determine which function of H/ACA RNP complex plays a more important role in the initiation of hematopoietic failure in DC, we measured the telomerase activity and rRNA processing in both *dkc1* and *nola1* deficient fish.

**Figure 4:**
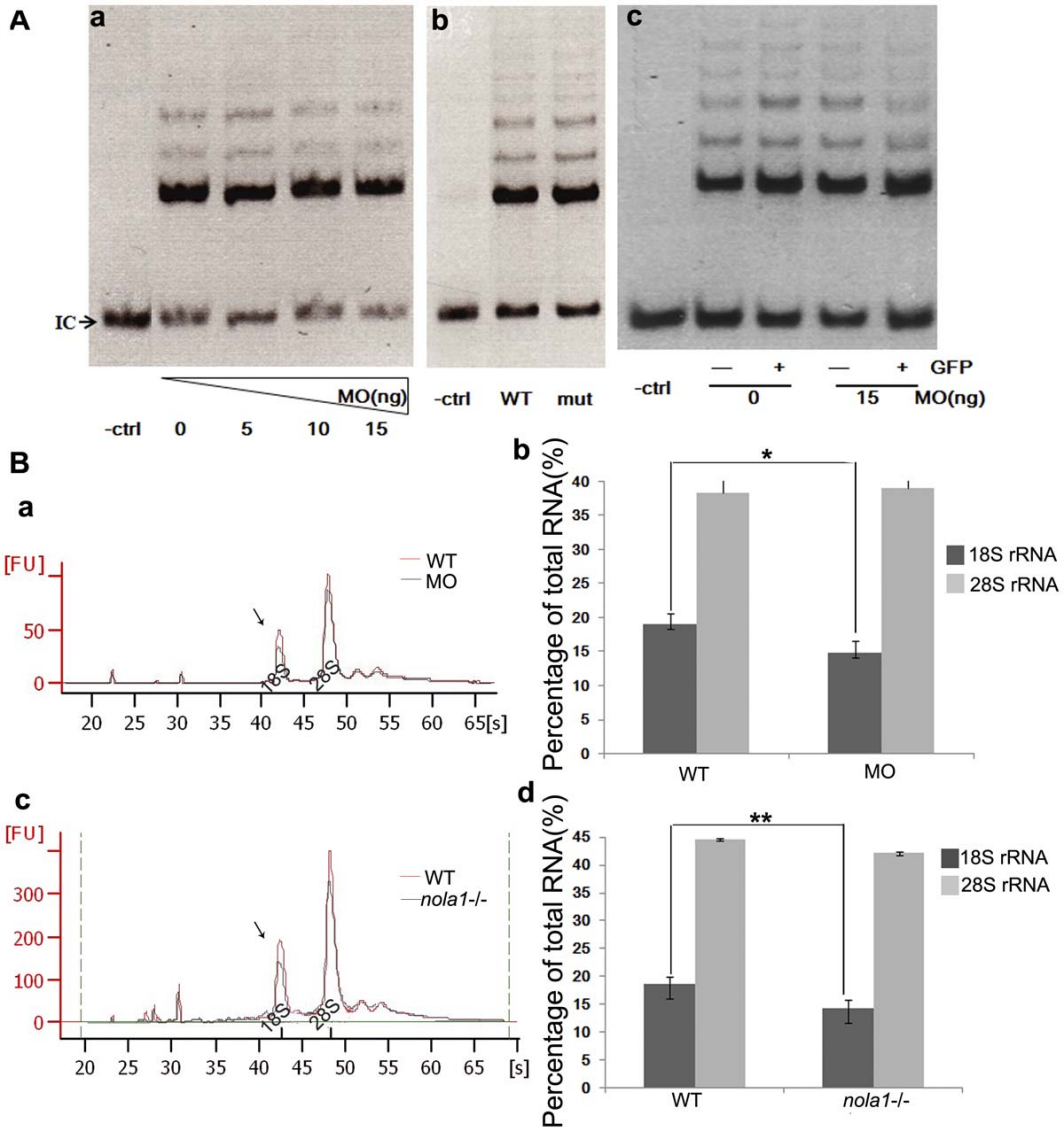
Analysis of telomere maintenance and rRNA processing in zebrafish *dkc1* and *nola1* defeicency. (**A**) TRAP Assay results showed no difference between *dkc1* morphants, *nola1* mutants and control embryos at 3 dpf when the mutant phenotype onset (**a** and **b**). (**c**) We injected *dkc1* MO into Tg(c-myb:GFP) fish. GFP-positive HSC were isolated by cell sorting (FACS) at 3 dpf. GFP positive (GFP+) and negative (GFP−) cells from control embryos (**0 ng MO**) and *dkc1* morphants (**15 ng MO**) were subjected to TRAP Assay. **IC**: Internal control; **-ctrl**: only lysis buffer but no embryo extracts added group. (**B**)Analysis of RNA processing showed that reduction of 18 S rRNA in *dkc1* morphant (**a** and **b**) at 48 hpf and in *nola1* mutant (**c** and **d**) at 3 dpf, but slight change or no change of 28 S rRNA. The average fold changes of percentage of 18 S rRNA and 28 S rRNA are represented by the bar graphs. **WT**: wild type control; **MO**: dkc1 morphant; **nola1−/−**: *nola1* mutant. ** indicates very significant changes at p<0.01, and * indicates significant changes at p<0.05 on the basis of independent Student *t* tests.

There was no significant difference in whole embryo lysate telomerase activity between morphants, mutants, and wild-type siblings at 3 dpf (Fig. 4Aa, b). To exclude the possibility of HSC-specific telomerase defects, we injected *dkc1* MO into one-cell stage of Tg(c-myb:GFP) fish embryos, FACS sorted the GFP positive cells, and measured their telomerase activity. No difference in telomerase activity was detected in sorted cells (Fig. 4Ac), suggesting that the hematopoietic failure seen in mutants/morphants is independent of telomerase function.

However, production of 18 S rRNA was defective in *dkc1* morphants at 48 hpf (Fig. 4Ba, b, arrow in a) prior to observation of the morphologic phenotype. In addition, *nola1* mutants showed approximately 25% less 18 S rRNA relative to total RNA at 3 dpf (Fig. 4B-c, d, arrow in c). There was no statistically significant change in production of 28 S rRNA. We obtained the same results from Northern blotting analysis using probes that were specifically designed to detect the internally transcribed sequence 1 (ITS1), internally transcribed sequence 2 (ITS2), and 18 S rRNA. Decrease of the rRNA precursor generating 18 S rRNA was observed in the lanes probed with ITS1 in both of *dkc1* morphant at 48 hpf and *nola1* mutant at 3 dpf compared with the wild type controls (arrows in Fig. S4B). Importantly, a significant reduction of total amount of 18 S rRNA was detected in the lanes probed with 18 S rRNA in both models. However, no significant difference was revealed in the lanes probes with ITS2, indicating that the processing of 28 S rRNA and 5.8 S rRNA appears to be normal or much less affected. Taken together, our data suggest that defects in rRNA processing, and not telomerase activity, may play the central role in DC patients' bone marrow failure.

## Discussion

The goal of this study is to reveal the cause of bone marrow failure in DC. The prevailing view is that DC is caused by telomere maintenance defects [23]. Indeed, mutations of several components of telomerase and shelterin complexes have been found in DC patients and in many cases telomere shortening is reported. However, not all data fit this notion. DKC1, NOP10, and NHP2 are involved not only in telomere maintenance but are also components of the H/ACA RNP complex. This conserved complex also performs site-specific pseudouridylation of ribosomal RNAs [24]. In a *Dkc1*-dificent mouse model, deregulation of ribosomal function and hematopoietic defects have been observed in early development while telomere shortening has been detected only in later generations [25]. Defects of rRNA maturation in a *Drosophila* mutant for an ortholog of this gene have also been reported [26]. In mouse *Dkc1*-deficient hepatocytes, accumulation of rRNA precursors and induction of *p53* pathway have been found [19]. *P53*-independent cell cycle arrest of cells depleted with dyskerin was also noted [27]. Based on findings described here, we believe that defects of ribosome biogenesis constitute an early event in the pathogenesis of DC and may be the major cause of bone marrow failure in this disease, although we cannot exclude the possibility that telomere shortening may contribute to DC at later stages. Our results agree with studies of Dkc1 mutant mice, which suggested that deregulated ribosome function is important for the initiation of DC [25]. These data may be relevant to some clinical cases of DC characterized by normal telomeres [28].

We found that the effect of deficiency of H/ACA RNP complex genes on hematopoiesis is mediated by *p53*. Defects in HSC development in *dkc1* and *nola1*-deficient zebrafish can be lessened by p53 inhibition. Zebrafish mutant for *nola1* has a phenotype similar to *dkc1* morphant including hematopoietic defects. Deficiency of both proteins results in *p53* up-regulation and the hematopoietic defects are rescued by *p53* inhibition. While this manuscript was in preparation, Pereboom et al reported that zebrafish mutation of *nop10*, another component of H/ACA RNP complex, caused similar p53 dependent phenotype due to RNA processing defect [29]. Therefore, our data together with previous reports reveal an important role for *p53* activation in mediating blood specific defects in DC as well as related diseases such as DBA [18]. Therapeutics targeting *p53* activation may have beneficial effects in treating these types of diseases.

Notably, mutations of GAR1/nola1 have not been reported in DC patients [16]. Earlier studies showed that H/ACA snoRNA accumulation did not require GAR1 [14], [30], [31]. However, recent data revealed a crucial role of Gar1/nola1 in the formation of functional H/ACA RNPs. First, Dyskerin forms a complex with Nop10, Nhp2, and Naf1, and then this complex binds the nascent precursor of H/ACA RNA to form inactive pre-H/ACA RNPs, which still lacks Gar1 [24]. The last step of H/ACA RNP assembly is the replacement of Naf1 with Gar1, which controls the transition from inactive pre-RNP to functional H/ACA RNP. Gar1/nola1 is highly conserved among species as illustrated by a successfully partial rescue of our zebrafish *nola1* mutant phenotype by injection of human *GAR1* mRNA. Given the key role of *Gar1* in H/ACA RNPs function, it is possible that its deficiency leads to early developmental defects such that *GAR1*-deficient human embryos do not survive in uteri. This situation may resemble the case of Diamond Blackfan Anemia. 9 ribosomal proteins have been found mutated in DBA. What is interesting that mutations in ribosomal proteins important for ribosomal biogenesis at early stages (such as RPS11), have never been found in humans presumably because their deficiency leads to severe defects incompatible with development. RPS19, most frequently mutated in DBA patients is involved in ribosome biogenesis only at later stages and its deficiency causes less damage. Yet zebrafish mutants for RPS11 and similar RPs have been recovered and their phenotypes are similar to mutants for RPs mutated in DBA. It may be that zebrafish embryos are more viable than mammalian embryos and may therefore provide models that cannot be created in mammalian species.

In the future, GAR1 should be taken into consideration for DC cases without mutations in the genes reported.

## Materials and Methods

### Ethics Statement

All animal experiments were approved by Institutional Animal Care and Use Committee (IACUC) of Peking University. The reference from IACUC of Peking University is LSC-ZhangB-1.

### Zebrafish Lines

Zebrafish (*Danio rerio*) were raised and maintained under standard laboratory conditions [32], [33]. Wild type fish lines were AB or Tuebingen (TU). *Nola1* mutant fish line was identified from an insertional retrovirus mutagenesis screen [20]. *P53* null fish were as described previously [34]. Transgenic fish Tg(c-myb:GFP) was generated by injecting a BAC construct containing *c-myb* regulatory sequences modified with *GFP* into one-cell stage wild type embryos. Founders and homozygous F_1_ were screened for *GFP* expression.

### Microinjection of morpholino oligonucleotides and mRNAs

Sequence of splicing *dkc1* MO (Gene Tools, Corvallis, OR, USA): 5′-GTTAATCCAAGACTTCATACCTGAC-3′. *P53* ATG MO was described previously [35]. Sequences of primers used for detecting the knockdown of *dkc1* in morphants are: *dkc1*RTF1: 5′-GAACATCAGGACGGCTCATT-3′ and *dkc1*RTR1: 5′-ATGCCCACATACTCTTTGCC-3′.

To make mRNA, coding sequences of *GAR1* and *nola1* were PCR amplified and cloned into the pCS2+ vector. mRNA were synthesized by in vitro transcription using mMESSAGE mMACHINE® SP6 Kit (Ambion) according to the manufacturer's instructions. Sequences of primers for cloning the coding sequences of *GAR1* and *nola1* are: *GAR1* RTF: 5′-CGGGATCCCACGGCTCAGCGTCAGGCAA-3′ and *GAR1* RTR: 5′-CCGCTCGAGAGCACCCACAGAGTGCCAGG-3′; *nola1* RTF2: 5′-CGGGATCCGCACGTGTGGTTCAGAGTCA-3′ and *nola1* RTR2: 5′-CCGCTCGAGTCCATGGTGGCAGCTGGAGT-3′.

### RNA isolation, reverse transcription and Real Time PCR

Total RNA was isolated using Trizol (Invitrogen, Carlsbad, CA, USA) from 30–40 embryos according to the manufacturer's instructions. cDNA was synthesized by reverse transcription using 2 ug RNA with Oligo(dT)_12–18_ (Invitrogen, Carlsbad, CA,USA). Real Time PCR was conducted using FastStart Universal SYBR Green Master (Roche, Diagnostics, Indianapolis, IN, USA) and a MyiQ Single-Color PCR thermal cycler (Biorad, Hercules, CA, USA). Each experiment was performed in triplicate and was repeated three times. Real Time PCR primers used for *p53* signaling pathway were described previously [36]. Sequences of Real Time PCR primers for *dkc1* and *nola1* were: *dkc1*RTF1: 5′-GAACATCAGGACGGCTCATT-3′ and *dkc1*RTR1: 5′-ATGCCCACATACTCTTTGCC-3′; *nola1* RTF1: 5′-AGGAGGCAGAGGTGGATTTA-3′ and *nola1* RTR1: 5′-CCCAGTGCGACAACATATTC-3′. *Dkc1* and *nola1* mRNA expression were normalized to *β-actin* using the method described previously [37] and compared to wild-type siblings.

### Whole-mount in situ hybridization

Whole-mount in situ hybridization was performed as described [38] using *c-myb*, *runx1*, *mpx*, *rag1* [36] and *nola1* riboprobes. Sequences of primers used for *nola1* probe synthesis were: *nola1*pRTF: 5′-GCACGTGTGGTTCAGAGTCA-3′ and *nola1*pRTR: 5′-ACGGAGCTGCCCAAAGAT-3′. *Runx1* expression analysis in *nola1* mutation was performed at 30 hpf. At this stage, the mutants were not distinguishable from wild type according to the morphology. After in situ hybridization, ∼25% (12/50) embryos exhibited the reduction of *runx1* shown as in figure 2 Bd, and ∼75% (38/50) showed the normal pattern as seen in figure 2 Bc. We confirmed those having expression shown in figure 2 Bd as *nola1* mutants and those in figure 2 Bc as wild type or heterozygous by PCR genotyping.

### Whole-mount o-dianisidine staining

Hemoglobin was detected by whole-mount o-dianisidine staining as described previously [36]. Briefly, 25 embryos under anesthesia were stained in o-dianisidine solution [(0.6 mg/ml o-dianisidine, 0.01 M sodium acetate (pH 4.5), 0.65% hydrogen peroxide, 40% ethanol)] for 15 min in the dark followed by two washes with PBS.

### Isolation of hematopoietic stem cells and FACS analysis

300 transgenic zebrafish embryos expressing GFP under *c-myb* promoter were dechorionated with protease at 3 days post fertilization (dpf) and washes with Ca^2+^ free Ringer's solution for 15 min. Embryos were incubated with 0.25% Trypsin-EDTA at 28 degrees Celsius for 30–50 min and then centrifuged at 300 g for 5 min. Cells were washed once with suspension solution [Leibovitz medium L-15, 0.8 Mm CaCl2, 1% FBS] and then resuspended in suspension solution. Suspension was filtered through a mesh capped tube. FACS sorting was performed with a flow cytometer sorter (BD AriaII). GFP positive and negative cells were sorted out.

### In vitro measurement of telomerase activity

The TRAPeze® telomerase detection kit (Millipore) was used according to the manufacturer's instructions. The PCR products were separated with 10% polyacrylamide gels and visualized by ethidium bromide staining.

### Analysis of RNA processing

RNA was isolated from 30 embryos at different stages (24 hpf, 48 hpf and 3 dpf) using Trizol (Invitrogen, Carlsbad, CA,USA) and cleaned up with Qiagen RNeasy Mini Kit (Qiagen,Valencia, CA, USA) according to the manufacturer's instructions. RNA samples were analyzed using Agilent RNA 6000 Nano Kit (Agilent, Waldbronn, Germany) according to the manufacturer's instructions.

### Northern blotting analysis

Total RNA was isolated from 50 embryos at 48 hpf for *dkc1* or 3 dpf for *nola1* using Trizol (Invitrogen, Carlsbad, CA, USA) and cleaned up with Qiagen RNeasy Mini Kit (Qiagen, Valencia, CA, USA) according to the manufacturer's instructions. Northern blots were operated as previously described [39]. Total RNA (1.0 µg per lane) was separated by electrophoresis on a 2% formaldehyde, 1.2% agarose gel and blotted onto a nylon membrane (Hybond-N, Amersham Biosciences). RNA blots were cross linked to the membrane by UV irradiation and probed with DIG labeled ITS1, ITS2 and 18 S rRNA probes using prehybridization and hybridization solution (Roche, Diagnostics, Indianapolis, IN, USA) at 65°C. The intensity of staining relative to the background was measured using ImageJ program.

## Supporting Information

Figure S1 **Temporal and spatial expression pattern of** ***nola1*** **.** (**A**) Semi-quantitative PCR result showed that *nola1* was expressed from about 2 hpf. (**B**) Non-specific expression of *nola1* was detected during the early stage of zebrafish development (**a**, **b**). Afterward, *nola1* expression was mainly detected in the brain and some inner organs. **a**: *nola1* expression at 7 hpf; **b**: *nola1* expression at 10 hpf; **c** and **c′**: *nola1* expression at 4 dpf. **a**, **b**: lateral view; **c**: lateral view with anterior to the left; **c′**: dorsal view with anterior to the left.
(TIF)

Figure S2 ***Nola1*** **is highly conserved among different species.** (**A**) Analysis result using Clustal W software showed protein sequences of *nola1* from different species have high similarity. (**B**) Synteny analysis data show the evolutionary conservation of *nola1*. (**C**) Microinjection of *GAR1* mRNA can partially rescue the mutant phenotype of *nola1* homozygous mutants at 5 dpf (red arrow and arrowhead in **a**, **b** and **c**). **a**: wild type sibling; **b**: *nola1* homozygous mutant; **c**: *nola1* homozygous mutant injected with human *GAR1* mRNA. All of the pictures of embryos are lateral view with anterior to the left.
(TIF)

Figure S3 **Rescue of hematopoietic defects of** ***nola1*** **mutant in** ***p53*** **mutant background.** (**A**) *p53* ^+/+^ *nola1* ^+/?^; (**B**) *p53* ^+/+^ *nola1* ^−/−^; (**C**) *p53* ^−/−^ *nola1* ^−/−^. Number of HSC (marked by *c-myb*, red arrows) was partially rescued in *nola1* and *p53* double mutant at 3 dpf.
(TIF)

Figure S4 **Deficiencies of both** ***dkc1*** **and** ***nola1*** **lead to the defects of rRNA processing.** (**A**) Schematic figure modified from previous report [29] shows the overview of rRNA processing. (**B**) A significant decrease of a precursor strand generating ITS1 and 18 S rRNA was detected in the lanes probed with ITS1 in both *dkc1* morphant and *nola1* mutant compared with wild type controls (arrows in **a** and **b**). The total amount of 18 S rRNA was reduced significantly as shown in the lanes probed with 18 S rRNA probe. The intensity of staining of 18 S rRNA relative to the background was measured using ImageJ program. In contrast, no obvious difference was detected in the lanes probed with ITS2 probe.
(TIF)
