# Supplementary figures and images for: Zebrafish Models for Dyskeratosis Congenita Reveal Critical Roles of p53 Activation Contributing to Hematopoietic Defects through RNA Processing
Source: PLoS One. 2012 Jan 27;7(1):e30188. doi: 10.1371/journal.pone.0030188 (PMC3267717; doi:10.1371/journal.pone.0030188)

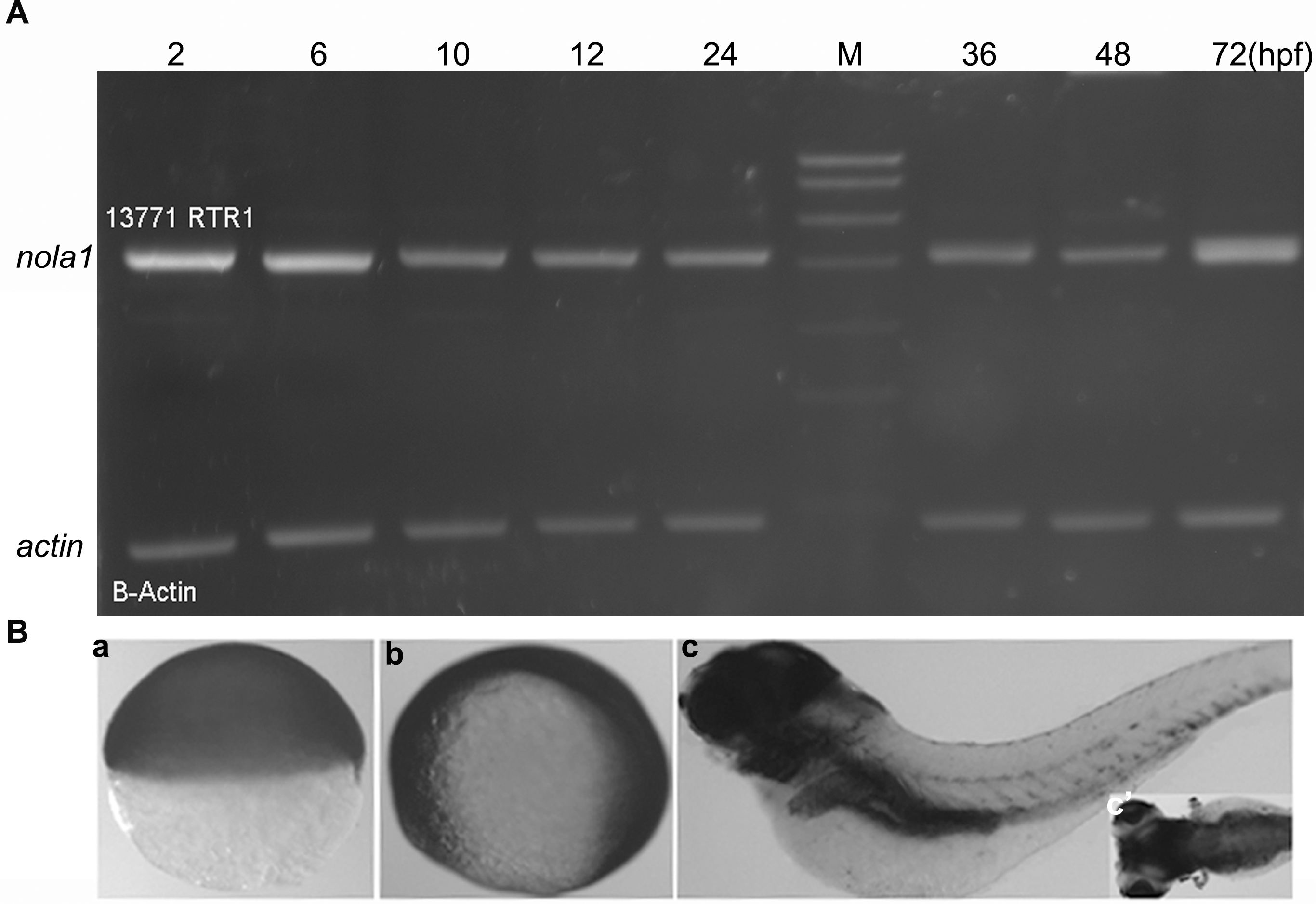

Supplement: Figure S1 — Temporal and spatial expression pattern of nola1 . (A) Semi-quantitative PCR result showed that nola1 was expressed from about 2 hpf. (B) Non-specific expression of nola1 was detected during the early stage of zebrafish development (a, b). Afterward, nola1 expression was mainly detected in the brain and some inner organs. a: nola1 expression at 7 hpf; b: nola1 expression at 10 hpf; c and c′: nola1 expression at 4 dpf. a, b: lateral view; c: lateral view with anterior to the left; c′: dorsal view with anterior to the left. (TIF) [file pone.0030188.s001.tif]

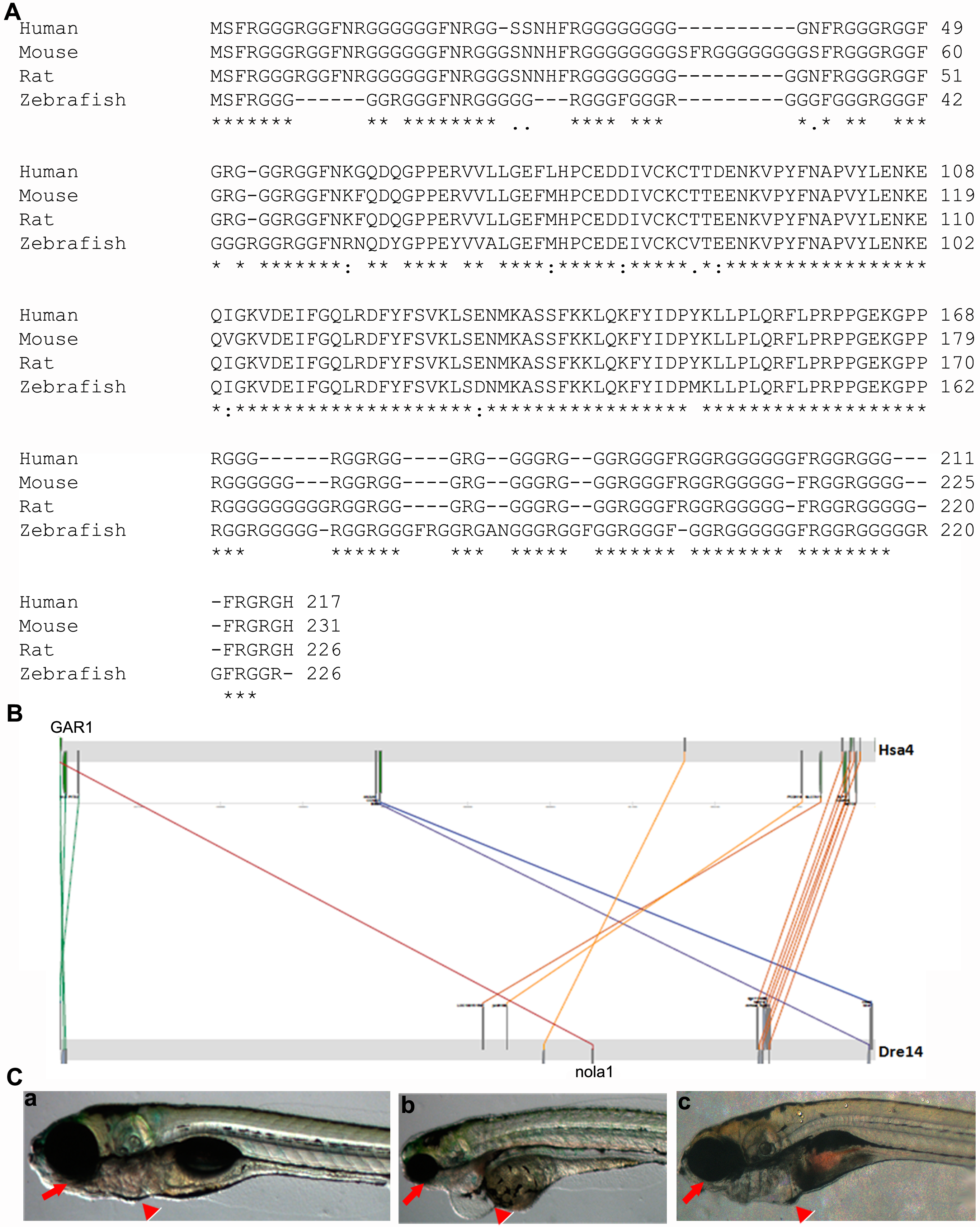

Supplement: Figure S2 — Nola1 is highly conserved among different species. (A) Analysis result using Clustal W software showed protein sequences of nola1 from different species have high similarity. (B) Synteny analysis data show the evolutionary conservation of nola1. (C) Microinjection of GAR1 mRNA can partially rescue the mutant phenotype of nola1 homozygous mutants at 5 dpf (red arrow and arrowhead in a, b and c). a: wild type sibling; b: nola1 homozygous mutant; c: nola1 homozygous mutant injected with human GAR1 mRNA. All of the pictures of embryos are lateral view with anterior to the left. (TIF) [file pone.0030188.s002.tif]

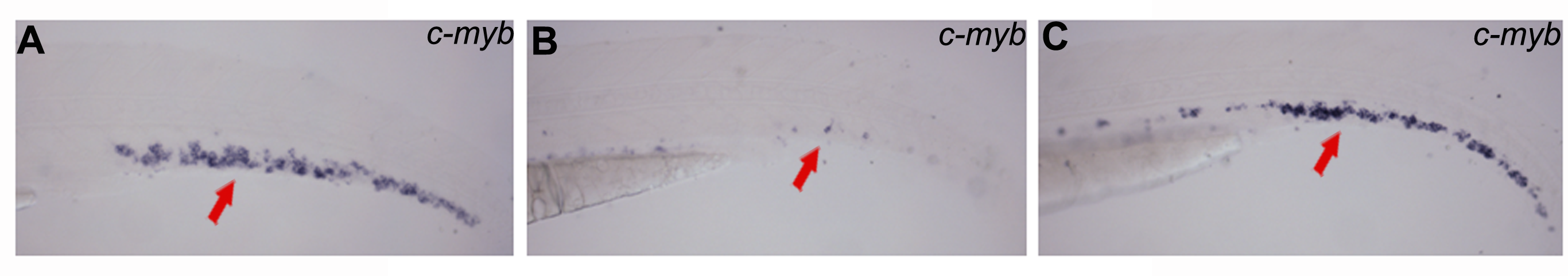

Supplement: Figure S3 — Rescue of hematopoietic defects of nola1 mutant in p53 mutant background. (A) p53 +/+ nola1 +/?; (B) p53 +/+ nola1 −/−; (C) p53 −/− nola1 −/−. Number of HSC (marked by c-myb, red arrows) was partially rescued in nola1 and p53 double mutant at 3 dpf. (TIF) [file pone.0030188.s003.tif]

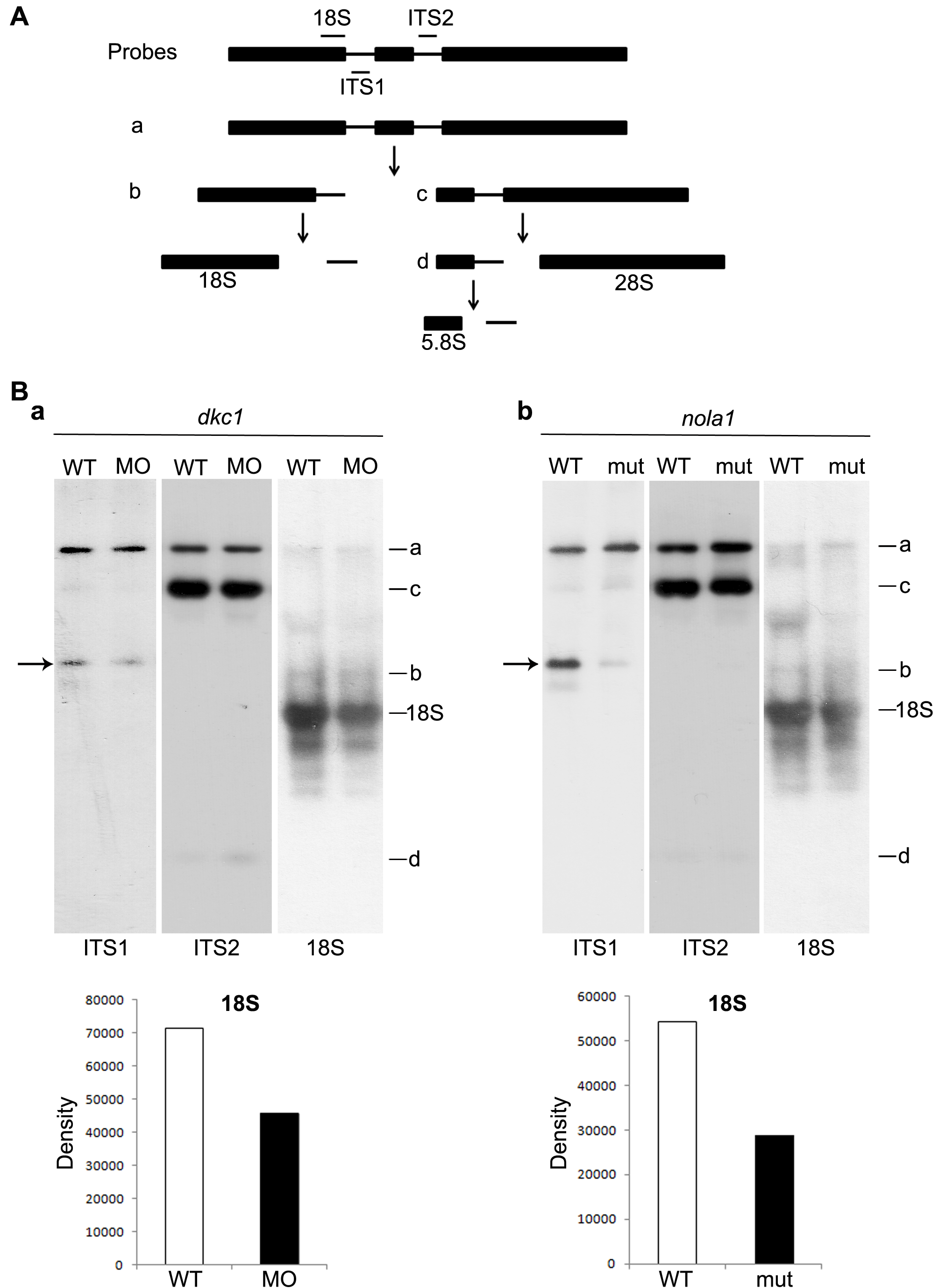

Supplement: Figure S4 — Deficiencies of both dkc1 and nola1 lead to the defects of rRNA processing. (A) Schematic figure modified from previous report [29] shows the overview of rRNA processing. (B) A significant decrease of a precursor strand generating ITS1 and 18 S rRNA was detected in the lanes probed with ITS1 in both dkc1 morphant and nola1 mutant compared with wild type controls (arrows in a and b). The total amount of 18 S rRNA was reduced significantly as shown in the lanes probed with 18 S rRNA probe. The intensity of staining of 18 S rRNA relative to the background was measured using ImageJ program. In contrast, no obvious difference was detected in the lanes probed with ITS2 probe. (TIF) [file pone.0030188.s004.tif]
